# Empowering Equitable Data Use Partnerships and Indigenous Data Sovereignties Amid Pandemic Genomics

**DOI:** 10.3389/fpubh.2021.742467

**Published:** 2021-11-11

**Authors:** Rodney C. Haring, Jessica W. Blanchard, Josephine D. Korchmaros, Justin R. Lund, Emily A. Haozous, Josie Raphaelito, Maui Hudson, Krystal S. Tsosie

**Affiliations:** ^1^Center for Indigenous Cancer Research, Roswell Park Comprehensive Cancer Center, Buffalo, NY, United States; ^2^Center for Applied Social Research, University of Oklahoma, Norman, OK, United States; ^3^Southwest Institute for Research on Women, University of Arizona, Tucson, AZ, United States; ^4^Department of Anthropology, University of Oklahoma, Norman, OK, United States; ^5^Pacific Institute for Research and Evaluation, Albuquerque, NM, United States; ^6^Te Kotahi Research Institute, University of Waikato, Hamilton, New Zealand; ^7^Vanderbilt University, Nashville, TN, United States; ^8^Native BioData Consortium, Eagle Butte, SD, United States

**Keywords:** Indigenous, American Indian/Alaska Native, COVID-19, genomics, Indigenous data sovereignty, data use agreements, broad consent, vaccine research

## Abstract

The COVID-19 pandemic has inequitably impacted Indigenous communities in the United States. In this emergency state that highlighted existing inadequacies in US government and tribal public health infrastructures, many tribal nations contracted with commercial entities and other organization types to conduct rapid diagnostic and antibody testing, often based on proprietary technologies specific to the novel pathogen. They also partnered with public-private enterprises on clinical trials to further the development of vaccines. Indigenous people contributed biological samples for assessment and, in many cases, broadly consented for indefinite use for future genomics research. A concern is that the need for crisis aid may have placed Indigenous communities in a position to forego critical review of data use agreements by tribal research governances. In effect, tribal nations were placed in the unenviable position of trading short-term public health assistance for long-term, unrestricted access to Indigenous genomes that may disempower future tribal sovereignties over community members' data. Diagnostic testing, specimen collection, and vaccine research is ongoing; thus, our aim is to outline pathways to trust that center current and future equitable relationship-building between tribal entities and public-private interests. These pathways can be utilized to increase Indigenous communities' trust of external partners and share understanding of expectations for and execution of data protections. We discuss how to navigate genomic-based data use agreements in the context of pathogen genomics. While we focus on US tribal nations, Indigenous genomic data sovereignties relate to global Indigenous nations regardless of colonial government recognition.

## Introduction

Indigenous communities continue to be disproportionately impacted by the COVID-19 (coronavirus disease 2019) pandemic. Incidence and age-adjusted mortality rates among American Indians and Alaska Natives (AI/AN) were, respectively, 3.5 times and 1.8 higher compared to non-Hispanic white persons across the United States (US) (1–3). Recent reports from August 2021 show a 600% increase in new cases among Native Hawaiians in Hawai'i, underscoring impacts of the latest surge for US Indigenous communities (4). Disparities in COVID-19 rates among Indigenous people are rooted in contemporary social and health inequities, including increased prevalence of underlying conditions, structural barriers to accessing resources for curbing viral transmission (i.e., clean water, personal protective equipment), underfunded tribal health systems, and geographic rurality (5, 6)—which are rooted in colonialism and complex histories of tribal-trust treaty relationships (7). Though apparent, the true extent of COVID-19 disparities among Indigenous people is subject to underreporting which may impede public health initiatives (8) related to biological testing and vaccination.

Amid the emergent conditions of the pandemic, concerns arise that Indigenous nations traded short-term needs for COVID-19 testing, surveillance, and vaccination with long-term, unrestricted access by non-tribal entities to Indigenous peoples' genomes which may undermine Indigenous data sovereignties. While some call for increased collection of biological data from Indigenous populations to understand the extent of COVID-19 disease burden disparities (9), we as Indigenous health researchers and non-Indigenous allies remind that extraction of any data from tribal nations without attribution to Indigenous data sovereignties can be equivalent to past research harms. Therefore, in expanding others' recognition of Indigenous sovereignties related to tribal public health responses (9–11), we outline a framework for partnering with Indigenous nations to ensure that genomic and other biological information collected from Indigenous individuals in pandemic crises–as part of diagnostic and antibody testing, clinical trial initiatives, and vaccine research–can benefit future Indigenous data sovereignties.

## Indigenous Genomic Data Sovereignties

Indigenous data sovereignties are defined as the “rights and interests of Indigenous peoples relating to the collection, ownership, and application of data about their people, lifeways, and territories” (12). When referring specifically to data derived from a part or whole of Indigenous peoples' genomes, we use the term “Indigenous genomic data sovereignties” (13). Settler-colonial recognition of these sovereignties are usually limited to, in the US context, the 574 federally recognized tribes via “nation-to-nation” policies. However, we recognize that data sovereignties are intrinsic to Indigenous peoples' right to self-govern (14) and must therefore extend beyond colonially-defined arbitrations of geographic state to include urban-displaced citizens of tribal nations and Indigenous groups of special and/or unrecognized status. Further, while some approaches to collecting data from Indigenous peoples try to leverage “individual vs. group” dynamics as a means of circumventing Indigenous genomic data sovereignties (13, 15), it is up to the communities to define data access and use of biological and genomic information collected from their people. While we use community-engaged models as a basis for the suggestions on equitable data use and sharing, we argue for a more empowered approach that centers Indigenous data decision authorities (14) first and foremost.

The assertion of Indigenous governances to self-determine public health initiatives for their own people (5) brought swift changes in COVID-19 incidence rates for some tribal nations. Sometimes in stark contrast to states' responses early in the pandemic, tribal nations—in particular but not limited to Arizona (16), Montana (17), North Dakota (18), and South Dakota (19, 20)—effectively led pandemic responses by implementing local mitigation strategies, restricting travel, mandating curfews and masks, creating culturally-tailored health messaging, and instituting contact tracing within their jurisdictions. Some states achieved greater equity in vaccine distribution among AI/AN populations (3, 21). Early efforts by tribes to vaccinate their populations initially led to high, though currently stagnating (22), rates of vaccination in many tribal communities, with nearly 70% vaccination rates for eligible individuals reported for the Meskwaki Nation (Sac and Fox Tribe of the Mississippi) (23), the Navajo Nation (24), among others (25).

While these measures certainly contribute to decreasing viral transmission, many tribal nations are still ill-equipped to provide diagnostic and antibody testing to confirm, trace, and treat COVID-19 cases. Considering these pre-existing deficiencies in tribal public health infrastructures that only exacerbate the need for local testing, many tribal nations rely on federal and municipal government services, University researchers, and private companies to conduct health data and biospecimen collection for diagnostic and antibody testing, clinical trials, and vaccine research. Collected data includes tribal group identifiers, identifiable data from kin, demographic information, and specimen data from which human genomic information can potentially be derived.

The emergency urge to quickly develop and disseminate a COVID-19 vaccine also brought forth many questions related to the pace of the Operation Warp Speed Vaccine Initiative, a public-private partnership between the US government and commercial entities. The “all-in” commercial investment strategy incentivized multiple vaccine developers to scale up manufacturing and distribution prior to completion of clinical studies (26), which brought an unprecedented rapidity to clinical trialing. There were concerns among tribal public health entities that vaccine companies and researchers were rushing tribal approval procedures or recruiting tribal citizens residing outside of their tribes' jurisdictions (27). There are sustained concerns that the vaccine research overly emphasize a Western ethic of individual informed consent when recruiting Indigenous individuals, particularly those who reside in urban city centers outside of tribal research protections, that is culturally inconsistent with Indigenous communitarian ethics and has potential to biologically re-identify Indigenous groups (15). More salient, however, is the persistent worry that Indigenous community members are broadly consenting (28) to ungoverned future use and ownership of Indigenous genomic data under the pretense of proprietary domain which may lead to co-optation and commercial exploitation (29). Compounding these concerns about the impacts of commercial interests are the ethical impacts of promoting the inclusion of Indigenous individuals in clinical trial research in ways that favor the goals of research institutions over tribal nations' sovereign rights to guarantee data governance and research benefit (30).

As a note, it is important to distinguish US Indigenous communities' relative lack of “vaccine hesitancy” for implementing a federally approved vaccine vs. the hesitation by many tribes to sign on as research trial participants prior to approval. There are distrusts related to the history of medical experimentation in Indigenous communities (31) that must be considered separately and distinctly from implementing vaccine programming after release to the general public.

## Need For Equitable Data Use Agreements

Data Use Agreements (DUAs) are legally binding contracts that stipulate terms related to limited transference of restricted data from one entity to another, to include procedures of data sharing, data access, and data licensing. Although entities involved should participate in developing and enforcing DUAs, tribal nations–as owners and stewards of data collected from tribal members– should be empowered as decision-makers in agreements involving data from their communities. In university-sponsored research, it is usually the institution that reviews and ensures compliance of DUAs according to funding regulations. For federally-funded data collection, often DUAs favor deposition of data into a central repository under the ethic of open or communal research use which can incongruent with Indigenous data sovereignties (14). Since federal data repositories are outside the governance and oversight of tribes, there have been tensions in negotiating the need to protect and recognize Indigenous data sovereignties while still supporting research collaborations (32). DUAs drafted by private organizations or commercial entities may also have corporate liability protections and intellectual property terms that favor those organizations. In any case, there is usually an inequitable power dynamic in that DUAs are drafted in the perspective of those empowered to collect and store data. Until tribal nations can create their own institutions for data collection and storage, which can take many years, unfortunately tribes will likely be disempowered to represent their data concerns (33). Furthermore, tribal nations may likely not be prepared to respond as quickly as needed to represent their data concerns during times of public health crises, as underscored during the events of the current and ongoing pandemic. Therefore, the need to address these inequities will continue to persist, and it is important to push for implementation of more responsible data use practices now.

The disproportionate impact of the pandemic and the need for crisis aid place Indigenous communities in a vulnerable position to forego critical review of DUAs by tribal research governances; additionally, tribes that do not already have these data oversights are further disenfranchised. Thus, there is urgency for developing and implementing data sharing practices that best serve Indigenous communities, particularly as post-pandemic activities (such as testing, vaccination, and research) must be continued for future public health. This urgency does not preclude the need to carefully co-develop terms of a DUA, which can have sustained impacts even after the pandemic state. Developing DUAs with shared understanding of expectations and execution of current and future data protections remains a critical component of equitable partnerships.

By recognizing the legacies of research harms associated with data, potential partners are more likely to be successful in practicing ethical research methods and avoiding future legal conflicts. Part of this process also entails respecting Indigenous data sovereignties related to the collection, use, storage, and oversight of Indigenous biological samples. To think transformatively about ameliorating health disparities will entail looking beyond genomic differences, especially as COVID-19 disparities are more proximally related to structural barriers to health than between-group biological differences. Thus, public health practitioners should be looking to long-term initiatives related to economic resiliency, public health leadership, and clinical and research practices—including macro-level data practices and clinical biospecimen collection and informatics.

## Framework Guidelines

### Respect and Collaboration Early in Negotiations

Creating DUAs empowers Indigenous communities as partners in the pandemic response. With sound DUAs, Indigenous communities are invited in planning conversations with potential partners to establish mutual understanding and respect that have impacts for future research. Furthermore, a DUA enables tribal nations to provide guidance on program implementation and any research products resulting from pandemic samples conducted within Indigenous landscapes. Crafting DUAs fosters discussions that could illuminate and address potential assumptions and differences in understandings before these challenges arose. If there is no clarification and resolution of issues through the DUA development, the research process can halt or another partner found to minimize any potential harms from the collection of contested data. This speaks to the importance of creating a DUA early in the process such that substantial time and resources are not devoted when a respectful and transparent partnership is unattainable.

External organizations invited to assist with pandemic response should recognize the Indigenous communities' ownership of their genomic data and show respect for local community members by involving them in the entire pandemic response, including the development, selection, analysis, presentation, and dissemination of any genomic data—whether internally, publicly, or in scientific realms. Indigenous citizen professionals working in the community engaged as part of the research can enrich the communities' understanding of the DUA processes. Community members and professionals should serve as integral members of pandemic response teams from start to finish (34).

### Specificity of Terms Is Key to Trust-Building

Tribal nations have sovereign and legal agency to self-direct their own pandemic response initiatives (35). DUAs should thus include an agreement of access for outside entities to operate in Indigenous communities. Access to what kinds of data is a key component of an access agreement (36). Access terms should specify locations, populations, records, and time frames. Though external organizations may be permitted access to certain tribal areas and populations, they should not assume they have *carte blanche* to collect any data in any form without consent. In multi-tribal partnerships, organizations should expect to establish DUAs with each Indigenous community separately with the knowledge that each community may have their own processes, priorities, foci, and expectations.

Access agreements should also detail personnel and their respective levels of data access and security (37–40). In this way, researchers can become trusted entities by which their cultural competencies, work ethics, and trustworthiness become known to tribal partners. When specific project personnel cannot be identified by name–for example, support personnel such as subcontractors or volunteers–partners should specify any credentials or qualifications (i.e. any ethical and cultural training or experience working with tribal nations) (41) of those individuals accessing Indigenous data.

Conducting emergency pandemic responses may result in unplanned access to aspects of community life and cultural practice, such as healing ceremonies, that might otherwise not be accessible to those outside of the community. External partners should by respectful and refrain from collecting any data or biological samples outside of expressed permissions. While some of these terms may be specified in research informed consent, not all public health data constitutes research. Therefore, creating access agreements between tribal nations and partners can illuminate these restrictions and increase understanding of collective goals and expectations for the rapid pandemic response and long-term collection, use, and storage of associated data.

Overall, access agreements are only one part of a macro-level data use agreements (29, 42–44). Another critical part is an agreement of for what and how the data can be used. Tribal nations should have sovereign data governance and intellectual property rights for technologies resulting from data collected on sovereign lands (44). DUAs should also indicate what will be shared, in what manner, and with whom (43). Outside entities partnering with tribal nations during pandemic times might also have specific goals regarding data-sharing, reporting measures, selling for profit, and should inform the tribal nation of any intentions to publish in journals, present at conferences, or commercialize tribal data and samples (45). A common component of DUAs is tribal right to review all dissemination products prior to publication, including press releases, manuscripts, presentations, and other reports that include data specific to their community.

### Good Data Stewardship Entails Safeguarding

Partner tribal nations should also be informed about how confidential information and samples will be protected and degrees of confidentiality from now and into the future (41). It should be the goal of the DUA to only report aggregate data, not individual data, in the report back to the tribal nation.

An important DUA safeguard is the review of jurisdiction and legal procedures early in implementation as a safety measure for both parties. External researchers and organizations should be aware that tribal nations may include clauses for the withdrawal from the contract. This is often done by tribal nations to prevent the release of sensitive information that misrepresents or stereotypes Indigenous peoples, or sensitive information that may harm the health, safety, or welfare of the communities or environment involved. It is also the case that tribal nations may stipulate that legal jurisdiction of procedures occurs in tribal court systems and that the contractual teams may be assessed fines for misconduct if harm, fraud, or unethical behavior is discovered.

It is the responsibility of both the tribal nations and the external researchers and organizations to ensure adherence to the DUA. Tribal nations are data owners and stewards, and they need to monitor who has access to the data and guide interpretations of research findings to ensure appropriate representation of tribal communities. As data users, external entities are responsible for ensuring adherence to the DUA or risk dissolution of partnerships with tribes. They should actively engage in ongoing assessment of their procedures related to data storage, use, and sharing to ensure continued adherence to the DUA regardless of staff turnover, changes in business practices and tools, and time.

### Building Sustainable Relationships

Creating DUAs will help build positive relationships between external research and organizations and the tribal nations through the process of creating equitable agreements and following an ethical framework (30, 42, 46–48). Sustained positive relationship building between these entities and respective tribal nations throughout the pandemic response is a continued step for business-to-community relationship building with tribal nations. Relationships require time to develop, strengthen, and build sustenance—even during uncertain and challenging times. The DUA process of relationship development is imperative because of historical mistrusts in Indigenous communities. Further, Indigenous culture and ways of knowing cannot be understood through brief emergency interaction during a public health crisis. Thus, tribal nations might view external entities who disengage in partnership development once access permission is granted as inauthentic, feigning to act in the best interest of Indigenous peoples, and disrespecting Indigenous sovereign public health. The most successful partnerships are those that are initiated early and actively work toward developing and strengthening the DUA-guided relationships throughout the duration of pandemic response and beyond.

## Future Implications

Pandemic genomic investigations in tribal nations are likely to evolve as nations adopt their own tribal biorepositories, storage procedures, and template DUA language. The ability of tribal nations themselves to generate and analyze this genomic information will empower them to be creative in the development of research agendas or revisiting with entities to negotiate existing secondary pandemic related genomic data. Further, maintaining DUAs, access, and control over pandemic genomic data will be supported by emerging aspirations to realize Indigenous data sovereignty (49). Therefore, wherever possible, we hope that tribes will continue to increasingly exert their sovereignties in the space of data collection commensurate with outside entities' interest in Indigenous genomic data. It ultimately should be the tribes' responsibility to steward data decisions that concern their peoples, and it is up to partners to respect these tribal sovereignties in order to develop trust relationships. Although there are guidelines for ethical research conduct in Indigenous communities (30), there is not yet a gold standard framework that is specific to conducting this type of research and data collection in the context of pandemic genomics. Therefore, the perspectives we presented can be useful for navigating genomic-based DUAs in the pathway to creating clinical to research process that can be helpful for tribal nations, should another public health emergency emerge.

Ultimately, equitable and beneficial pandemic response in Indigenous landscapes honors Indigenous Knowledges and respects tribal sovereignties. Pandemic and post-pandemic genomic research with Indigenous communities is crucial and, when conducted respectfully, can provide guided direction for improved future societal wellness. As shared by Indigenous community leader Michael Martin, “Every action we take we have to be mindful seven generations up” (https://www.youtube.com/watch?v=YyuSc_jkG-s).

## Data Availability Statement

The original contributions presented in the study are included in the article/supplementary material, further inquiries can be directed to the corresponding author/s.

## Author Contributions

RH initiated the collaboration and wrote the first draft of the manuscript. JB, JL, JK, EH, JR, MH, and KT contributed substantial intellectual and writing contributions through multiple iterations of the manuscript. KT oversaw the writing collaboration and final drafts of the manuscript. All authors contributed to manuscript revision, read, and approved the submitted version.

## Funding

This research was supported by the National Human Genome Research Institute and the Center for the Ethics of Indigenous Genomics Research (RM1HG009042). Additional shared resources were supported by the National Cancer Institute via the Roswell Park Comprehensive Cancer Center (P30CA016056).

## Conflict of Interest

KT serves as a non-compensated board member of a non-profit 501(c)3 Indigenous biobank initiative, the Native BioData Consortium, in the United States. The remaining authors declare that the research was conducted in the absence of any commercial or financial relationships that could be construed as a potential conflict of interest.

## Publisher's Note

All claims expressed in this article are solely those of the authors and do not necessarily represent those of their affiliated organizations, or those of the publisher, the editors and the reviewers. Any product that may be evaluated in this article, or claim that may be made by its manufacturer, is not guaranteed or endorsed by the publisher.
